# The moderating role of partners’ education on early antenatal care in northern Ghana

**DOI:** 10.1186/s12884-022-04709-9

**Published:** 2022-05-05

**Authors:** Paschal Awingura Apanga, Maxwell Tii Kumbeni, James Kotuah Sakeah, Ayokunle A. Olagoke, Olufemi Ajumobi

**Affiliations:** 1 University of Nevada, Reno, School of Public Health, Reno, USA; 2 Oregon State University, College of Public Health and Human Sciences, Corvallis, USA; 3 University of Ottawa, School of Epidemiology and Public Health, Ottawa, Canada; 4 University of Illinois at Chicago, School of Public Health, Chicago, USA

**Keywords:** early antenatal care, partner, education, effect modification, Ghana

## Abstract

**Background:**

Early antenatal care (ANC) is essential for improving maternal and child health outcomes. The primary aims of this study were to 1) estimate the association between partners’ education attainment and early ANC, and 2) determine whether partners’ level of education modified the relationship between mothers’ education, mothers’ age, planned pregnancy, employment status and early ANC.

**Methods:**

Data were obtained from a cross-sectional study conducted from April to May 2021 among 519 mothers with a live birth in the past year in the Nabdam district in the Upper East Region in northern Ghana. Generalized estimating equations were used to assess whether partners’ level of education modified the relationship between mothers’ education, mothers’ age, planned pregnancy, employment status and early ANC. Effect modification was assessed on the additive and multiplicative scales using adjusted prevalence ratios (aPR) and corresponding 95% confidence intervals.

**Results:**

Mothers whose partners had secondary or higher education had a 26% higher prevalence of early ANC compared to mothers whose partners had less than a secondary level of education (aPR: 1.26, 95% CI: 1.05,1.51). There was evidence of effect modification by partners’ education on the relationship between planned pregnancy and early ANC on both the additive (Relative excess risk due to interaction [RERI]: 0.61, 95% CI: 0.07,0.99), and multiplicative (ratio of PRs: 1.64, 95% CI: 1.01,2.70) scales. Among mothers whose partners had less than secondary education, mothers who had teenage pregnancy (i.e., aged 18–19 years old during pregnancy) were less likely to have early ANC compared to those who did not have teenage pregnancy (aPR: 0.71, 95% CI: 0.53,0.97). Among mothers whose partners had a secondary or higher education, early ANC was more prevalent among employed mothers compared to those who were unemployed (aPR: 1.27, 95% CI: 1.02,1.57).

**Conclusions:**

Our findings suggest that whilst mothers whose partners had a secondary or higher education were more likely to initiate early ANC, supporting such women to plan their pregnancies can further increase the coverage of early ANC.

## Background

Antenatal care (ANC) is the routine care provided by skilled healthcare professionals to pregnant women and adolescent girls between conception and the onset of labour [1]. ANC presents a unique and lifesaving window of opportunity for skilled healthcare professionals to prevent, identify and manage pregnancy-related complications [2]. Access to quality ANC can contribute to significantly reduce maternal and perinatal morbidity and mortality [3]. The World Health Organization (WHO) in 2016 revised the focused ANC (FANC) model and increased the number of ANC visits from the minimum of 4 visits to 8 contacts as this will reduce perinatal mortality and provide a positive pregnancy experience for women [2, 4]. The current ANC model recommends that the first ANC contact should take place within the first 12 weeks of pregnancy followed by an additional seven contacts [2].

Early ANC is recommended at a gestational age of less than 12 week (i.e., first trimester of pregnancy) [5]. Its initiation and the quality of service provided has been emphasized in the revised ANC guidelines [2]. The timing of early ANC is paramount for skilled healthcare professionals to provide care and information to pregnant women in their first trimester of pregnancy. Skilled healthcare professionals also take the opportunity during this period to engage pregnant women in health promotion, prevention, screening, and disease detection [6]. Early ANC allows for pregnant women to be screened for congenital anomalies and supplementation of folic acid for prevention of neutral tube defects [7]. Screening and diagnostic tests are also conducted for sexually transmitted infections, anaemia and non-communicable diseases such as hypertension and diabetes and are treated when detected [7]. In addition, early ANC does not only allow for accurate dating of pregnancy in the first trimester, which is essential in planning for delivery [8, 9], but also provides a window of opportunity to deliver public health education and guidance on modifiable risk factors. These modifiable risk factors include alcohol consumption, smoking, drug abuse, malnutrition, obesity, and occupational exposures [10]. Despite the benefits of early ANC, the coverage of early ANC is 24% in low income countries [1], and 62% in Ghana [11].

Previous studies in low and middle-income countries have established several factors associated with early ANC. Women with a secondary or higher education were more likely to have early ANC compared to women with no formal education [12, 13]. Women whose pregnancies were planned had higher odds of early ANC compared to women whose pregnancies were unplanned [14]. Employed women and younger mothers were also more likely attend early ANC compared to unemployed women and older mothers respectively [13, 15, 16]. It was also recently reported that women whose husbands had secondary or higher education were associated with having early ANC compared to women whose husbands had no formal education [17]. However, it is not known whether partners’ level of education will modify the relationship between mothers’ education, mothers’ age, planned pregnancy, employment status and early ANC.

In Ghana, studies on early ANC are limited [13, 18]. It is also unclear if the level of a partner’s education can impact on a woman having early ANC. It is also unknown whether partners’ level of education will modify the relationship between mothers’ education, mothers’ age, planned pregnancy, employment status and early ANC. The primary aims of this study were to characterize the association between partners’ education and early ANC, and to assess whether partners’ level of education modified the relationship between mothers’ education, planned pregnancy, mothers’ age, employment status and early ANC on the additive and multiplicative scales.

## Methods

### Study population

This was a cross-sectional study conducted from April to May 2021 in the Nabdam district in the Upper East Region of Ghana. The study population comprised of mothers with a live birth within the past year. The inclusion criteria were mothers attending child welfare clinics in the Nabdam district and who were at least 18 years old at the time of the study.

### Study setting

The Nabdam district located in the Upper East region in northern Ghana has an estimated population of 51,861 [19]. The district lies between latitudes 10^0^ 47″ and 10^0^ 57″ north and longitudes 0^0^ 31″ and 1^0^ 15″ west. Approximately, 9995 women are of reproductive age and 970 deliveries were conducted in 2020 [20]. The district has an estimated 100 midwives and community health nurses who offer antenatal care across several healthcare facilities. These healthcare facilities include two clinics, four health centres and 18 Community-based Health Planning and Services (CHPS) compounds [20].

### Sampling strategy

We used a convenient sampling approach to sample mothers who met our inclusion criteria. Trained research assistants sampled mothers from child welfare clinics in the Nabdam district and administered structured questionnaires during clinic hours. Mothers responded to questions related to their socio-demographic and obstetric-related characteristics (e.g., parity, planned pregnancy, early ANC) when pregnant with their current child. Prior to data collection for our main study, the survey instrument was pre-tested on ten mothers with a live birth in the past year in Bolgatanga East district. Additionally, trained research assistants reviewed and extracted information from the maternal and child health record book, which contains records of some socio-demographic characteristics and obstetric indicators for the mother when she was pregnant.

### Sample size

The minimum sample size required for the study was estimated using Epi info version 7.1. The sample size was estimated with the assumption that 50% of pregnant women had early antenatal care since the prevalence of early ANC was unknown in the study setting (i.e., Nabdam district) [20]. The minimum sample size estimated was 407 (including a non-response rate of 10%) using a 5% margin of error with a 95% confidence interval. During data collection, 541 mothers were invited to participate, but 22 mothers were ineligible. Therefore, 519 mothers took part in our study.

### Primary outcome

The primary outcome was early ANC. Early antenatal care was defined as pregnant women who had antenatal contact with the healthcare provider at a gestational age of less than 12 weeks (i.e., within first trimester) [5]. Early antenatal care was assessed by reviewing the maternal and child health record book, which records the number of antenatal care contacts and the trimester of pregnancy when each contact was made with a healthcare provider when the mother was pregnant. The value of “1” and “0” were assigned to mothers who had early ANC and those who did not respectively.

### Exposures

The exposures of interest include mother and partners’ highest educational level (secondary or higher education, less than secondary), planned pregnancy (yes, no); mothers’ age (18–19 years, ≥ 20 years), and employment status (employed. unemployed). A partner was either a husband or a boyfriend to the mother when she was pregnant. Being employed was either formal or informal. Exposures such as mother and partners’ education, mothers’ age, mothers’ education, and employment status were self-reported measures assessed using the maternal and child health record book, whilst planned pregnancy was self-reported during the survey about their last pregnancy.

### Covariates

The covariates were: marital status (single, married); place of residence (urban, rural); health insurance status (insured, uninsured); and parity (≥ 4 children, 0–3 children). Other covariates include household size (i.e., a continuous variable) and whether the coronavirus 2019 (COVID-19) pandemic affected my ANC attendance (yes, no).

Marital status, place of residence and parity were assessed using the maternal and child health record book, whilst household size, health insurance status and whether COVID-19 pandemic affected my ANC attendance were self-reported during the survey. The variable selection for our study was guided on prior knowledge of existing literature [12, 13, 15, 17].

### Data analysis

Descriptive statistics were used to describe our study population. Characteristics of study participants were presented in proportions for categorical variables and mean and standard deviation for continuous variables.

We conducted our analyses with five generalized estimating equation (GEE) models to achieve our study objectives using adjusted prevalence ratios (aPRs) [11, 21, 22]. The first model was used to assess the relationship between partners’ education and early ANC, whilst controlling for mothers’ education, planned pregnancy, mother’s age, employment status, parity, marital status, place of residence, health insurance status, household size and whether COVID-19 pandemic affected a mother’s ANC attendance. All potential confounders were specified as *a priori*, as we believe there is a biological plausibility that these variables might be associated with both the exposure and the outcome of interest.

The second model was used to assess whether the relationship between a mother’s education and early ANC was modified by partners’ education by introducing an interaction term (mothers’ education*partners’ education) into the model. Effect modification was assessed on the multiplicative and additive scales, whilst adjusting for planned pregnancy, mother’s age, employment status, parity, marital status, place of residence, health insurance status, household size and whether COVID-19 pandemic affected a mother’s ANC attendance.

The third model was used to characterize whether the association between planned pregnancy and early ANC was modified by partners’ education by introducing an interaction term between planned pregnancy and partners’ education (planned pregnancy*partners’ education). Effect modification was also assessed on the multiplicative and additive scales, whilst controlling for mothers’ education, mother’s age, employment status, parity, marital status, place of residence, health insurance status, household size and whether COVID-19 pandemic affected a mother’s ANC attendance.

A fourth model assessed whether the association between a mother’s age and early ANC varies by the level of a partner’s education. We introduced an interaction term between mothers’ age and partners’ education (mothers’ age*partners’ education) into the model and effect modification was assessed on the multiplicative and additive scales, whilst adjusting for mothers’ education, planned pregnancy, employment status, parity, marital status, place of residence, health insurance status, household size and whether COVID-19 pandemic affected a mother’s ANC attendance.

The fifth model assessed whether the association between employment status and early ANC was modified by partners’ education on the multiplicative and additive scales. This was done by introducing an interaction term between employment status and partner’s education (employment status*partners’ education), whilst controlling for mothers’ education, planned pregnancy, mother’s age, parity, marital status, place of residence, health insurance status, household size and whether COVID-19 pandemic affected a mother’s ANC attendance.

Effect modification on the additive scale was assessed using the relative excess risk due to interaction (RERI) as this is the most appropriate additive measure, which is of public health importance [23–25]. The RERI and corresponding 95% confidence intervals (CIs) were assessed using the “MOVER” approach proposed by Zou [26]. RERI was presented using prevalence ratio to estimate the risk ratio as our outcome was common and risk could not be directly determined from our study. An estimate greater than zero signified positive effect modification, while an estimate less than zero signified negative effect modification [27].

Our results on effect modification were presented according to STROBE (Strengthening the reporting of observational studies in epidemiology) recommendations [28]. We also included results on effect estimates of our exposures across the strata of another factor (i.e., partners’ education) as recommended by Knol and VanderWeele [27]. The format we present our results will therefore allow readers to obtain sufficient information needed to assess effect modification [27]. The data analyses were conducted using SAS version 9.3 (SAS Institute, Cary, NC).

## Results

### Study sample

The study population was made up of 519 mothers with a live birth in the past one year. The mean age of mothers was 26 ± 6.7 years. Approximately, 54% of mothers had early ANC when they were pregnant. Many of the mothers (78%), and their partners’ (71.7%), educational level was less than secondary education. Majority of the mothers were unemployed (75.5%) and had many of their pregnancies planned (66.4%). Approximately, 22% of mothers had teenage pregnancy (i.e., pregnancy within the ages of 18–19 years) prior to delivery (Table 1).

**Table 1:** Characteristics of the study population (*n* = 519)

Variable	N (%) or Mean (SD)
Mother’s age (years)	26 (6.7)
Mother’s age	
≥ 20	407 (78.4)
18–19 years	112 (21.6)
Marital status	
Married	451 (86.9)
Single	68 (13.1)
Mother’s education	
Less than secondary education	404 (78.0)
Secondary or higher education	114 (22.0)
*missing*	1
Place of residence	
Rural	340 (65.5)
Urban	179 (34.5)
Employment status	
Unemployed	392 (75.5)
Employed	127 (24.5)
Health insurance status	
Uninsured	193 (37.2)
Insured	326 (62.8)
Partner’s education	
Less than secondary education	370 (71.7)
Secondary or higher education	146 (28.3)
*missing*	3
Household size	7 (3.0)
Parity	
0–3	450 (86.7)
≥4	69 (13.3)
Planned pregnancy	
No	174 (33.6)
Yes	344 (66.4)
*missing*	1
COVID-19 pandemic affected my ANC attendance	
No	489 (94.4)
Yes	29 (5.6)
*missing*	1
Early antenatal care	
No	239 (46.1)
Yes	280 (53.9)

### The association between partners’ education and early antenatal care

Mothers whose partners had a secondary or higher education had a 26% higher prevalence of early ANC compared to mothers whose partners level of education was less than secondary education (aPR: 1.26, 95% CI: 1.05,1.51).

Other factors such as mothers’ age during pregnancy and planned pregnancy were associated with early ANC. Mothers who were aged 18-19 years (i.e., teenage pregnancy) had 25% lower prevalence of early ANC compared to mothers who were older (aPR: 0.75, 95% CI: 0.59,0.96). Mothers whose pregnancies were planned had 1.36 times the prevalence of early ANC compared to mothers whose pregnancies were unplanned (aPR: 1.36, 95% CI: 1.09,1.69) [Table 2].

**Table 2:** The association between partners’ education and early antenatal care

Variable**	Unadjusted PR (95% CI)	Adjusted PR (95% CI)
**Main exposure of interest**		
Partner’s education*		
Less than secondary education	1	1
Secondary or higher education	1.32 (1.13,1.54)	1.26 (1.05,1.51)
**Potential confounders**		
Age*		
≥ 20	1	1
18–19 years	0.68 (0.53,0.87)	0.75 (0.59,0.96)
Planned pregnancy*		
No	1	1
Yes	1.54 (1.26,1.88)	1.36 (1.09,1.69)
Mother’s education		
Less than secondary education	1	1
Secondary or higher education	1.14 (0.95,1.36)	0.97 (0.80,1.19)
Employment status		
Unemployed	1	1
Employed	1.21 (1.03,1.43)	1.07 (0.90,1.27)
Place of residence		
Rural	1	1
Urban	0.96 (0.81,1.14)	0.89 (0.75,1.04)
Marital status		
Married	1	1
Single	0.59 (0.42,0.84)	0.82 (0.57,1.17)
Health insurance status		
Uninsured	1	1
Insured	1.13 (0.96,1.35)	1.01 (0.85,1.20)
Household size	1.00 (0.97,1.03)	1.00 (0.97,1.02)
Parity		
0–3	1	1
≥ 4	0.81 (0.62,1.07)	0.80 (0.60,1.06)
COVID-19 pandemic affected my ANC attendance		
No	1	1
Yes	0.89 (0.6,1.3)	0.85 (0.61,1.19)

**Model 1; *indicates statistically significant *P*-values <0.05 (i.e., two sided); PR = Prevalence ratio; 1 = Reference category

### Effect modification by partners’ level of education

The aPRs with CIs and *p*-values were presented for mothers with secondary or higher education only (aPR: 0.90, 95% CI: 0.60,1.34), for mothers whose partners had secondary or higher education only (aPR: 1.22, 95% CI: 0.99,1.50), and for mothers and their partners with secondary or higher education (aPR: 1.24, 95% CI: 1.03,1.50), where less than secondary education among mothers and their partners was the reference category. There was no evidence of effect modification on the additive (RERI: 0.12, 95% CI: −0.41,0.52), and multiplicative (ratio of PRs: 1.13, 95% CI: 0.72,1.79) scales (Table 3).

**Table 3:** Effect modification of the association between mothers’ education and early antenatal care by partners’ education

	Mother has less than secondary education		Mother has secondary or higher education		PR (95% CI) for mothers’ education within strata of partners’ education
	N with/without outcome	PR (95% CI)	N with/without outcome	PR (95% CI)	
Partner has less than secondary education	171/168	1.00 (Reference)	13/17	0.90 (0.60,1.34); *p = 0.596*	0.90 (0.60,1.34); *p = 0.596*
Partner has secondary or higher education	41/22	1.22 (0.99,1.50); *p = 0.060*	55/28	1.24 (1.03,1.50); *p = 0.026*	1.02 (0.81,1.28); *p = 0.886*

Measure of effect modification on additive scale: RERI (95% CI) = 0.12 (−0.41,0.52); *p = 0.618*

Measure of effect modification on multiplicative scale: ratio of PRs (95% CI) = 1.13 (0.72,1.79); *p = 0.583*

Model II: PRs are adjusted for age, marital status, place of residence, employment, health insurance, household size, parity planned pregnancy and COVID-19 pandemic

There was positive effect modification of planned pregnancy across the strata of partners’ education on the additive scale (RERI: 0.61, 95% CI: 0.07,0.99). There was also positive effect modification on the multiplicative scale (ratio of PRs:1.64, 95% CI: 1.01,2.70) [Table 4].

**Table 4:** Effect modification of the association between planned pregnancy and early antenatal care by partners’ education

	Unplanned pregnancy		Planned pregnancy		PR (95% CI) for planned pregnancy within strata of partners’ education
	N with/without outcome	PR (95% CI)	N with/without outcome	PR (95% CI)	
Partner has less than secondary education	55/77	1.00 (Reference)	128/109	1.19 (0.93,1.52); *p = 0.169*	1.19 (0.93,1.52); *p = 0.169*
Partner has secondary or higher education	14/26	0.84 (0.52,1.36); *p = 0.476*	82/24	1.64 (1.26,2.12); *p < 0.001*	1.95 (1.25,3.04); *p = 0.003*

Measure of effect modification on additive scale: RERI (95% CI) = 0.61 (0.07,0.99); *p = 0.010*

Measure of effect modification on multiplicative scale: ratio of PRs (95% CI) = 1.64 (1.01,2.70); *p = 0.033*

Model III: PRs are adjusted for age, marital status, place of residence, employment, health insurance, household size, parity mothers’ education and COVID-19 pandemic

There was no evidence of effect modification on either the additive or multiplicative scales by partners’ education on the association between mothers’ age and early ANC. However, among the category of mothers whose partners had less than secondary education, mothers aged 18–19 years old had 29% lower odds of having early ANC compared to mothers who were aged 20 years or older (aPR:0.71, 95% CI: 0.53,0.97) [Table 5]. The relationship between employment status and early ANC was also not modified by partners’ education on either the additive or multiplicative scales. However, among the stratum of mothers whose partners had a secondary or higher education, mothers who were employed were more likely to have early ANC compared to unemployed mothers (aPR: 1.27, 95% CI: 1.02,1.57) [Table 6].

**Table 5:** Effect modification of the association between mothers’ age and early antenatal care by partners’ education

	≥ 20 years		18–19 years (Teenagers)		PR (95% CI) for mothers’ age within strata of partners’ education
	N with/without outcome	PR (95% CI)	N with/without outcome	PR (95% CI)	
Partner has less than secondary education	153/132	1.00 (Reference)	31/54	0.71 (0.53,0.97); *p = 0.030*	0.71 (0.53,0.97); *p = 0.030*
Partner has secondary or higher education	83/38	1.22 (1.01,1.48); *p = 0.037*	13/12	1.02 (0.7,1.51); *p = 0.902*	0.84 (0.56,1.24); *p = 0.376*

Measure of effect modification on additive scale: RERI (95% CI) = 0.09 (−0.37,0.60); *p = 0.742*

Measure of effect modification on multiplicative scale: ratio of PRs (95% CI) = 1.17 (0.72,1.91); *p = 0.537*

Model IV: PRs are adjusted for planned pregnancy, marital status, place of residence, employment, health insurance, household size, parity, mothers’ education, and COVID-19 pandemic

**Table 6:** Effect modification of the association between employment status and early antenatal care by partners’ education

	Unemployed		Employed		PR (95% CI) for employed within strata of partners’ education
	N with/without outcome	PR (95% CI)	N with/without outcome	PR (95% CI)	
Partner has less than secondary education	142/148	1.00 (Reference)	42/38	0.95 (0.74,1.22); *p = 0.695*	0.95 (0.74,1.22); *p = 0.695*
Partner has secondary or higher education	59/40	1.16 (0.94,1.43); *p = 0.177*	37/10	1.47 (1.17,1.84); *p = 0.001*	1.27 (1.02,1.57); *p = 0.034*

Measure of effect modification on additive scale: RERI (95% CI) = 0.36 (−0.02,0.71); *p = 0.058*

Measure of effect modification on multiplicative scale: ratio of PRs (95% CI) = 1.33 (0.96,1.83); *p = 0.078*

Model V: PRs are adjusted for planned pregnancy, marital status, place of residence, parity, health insurance, household size, Mothers’ age, mothers’ education, and COVID-19 pandemic

## Discussion

The main objectives of this study were to estimate the association between partners’ education and early ANC, and to assess whether partners’ level of education modified the relationship between mothers’ education, planned pregnancy, mothers’ age, employment status and early ANC on both the additive and multiplicative scales. Our findings suggests that early ANC was more prevalent among mothers whose partners had secondary or higher education compared to mothers whose partners had less than secondary education. We also found that the relationship between planned pregnancy and early ANC was modified by partners’ education on both the additive and multiplicative scales. Our findings also revealed that among mothers whose partners had less than a secondary education, mothers who had teenage pregnancy were less likely to have early ANC compared to mothers who were 20 years or older. Among mothers whose partners had a secondary or higher education, early ANC was more prevalent among employed compared to unemployed mothers.

Our findings on early ANC more prevalent among mothers whose partners had a secondary or higher education may be suggestive of several reasons. Males with secondary or higher educational attainment are associated with living in wealthier households in Ghana [29]. Therefore, mothers whose partners are wealthier may provide financial support to their wives to initiate early ANC compared to mothers whose partners had less than a secondary education. Such financial support can make a difference as travel cost and other incidental expenses have been reported as barriers to accessing ANC including early ANC in Ghana [13, 30, 31]. Mothers whose partners had secondary or higher education are also more likely to be knowledgeable about maternal health services including early ANC compared to mothers whose partners had less than a secondary education. Mothers whose partners had secondary or higher education were better informed and communicate well with their wives about the importance of ANC and may also provide more autonomy to their wives to make reproductive health decisions [32–35]. Our finding was similar to a previous study that found higher odds of early ANC among women whose husband’s had secondary or higher education [17], however, this study was limited to only married women. The finding in our study reflects the important role partners’ education may play in promoting early ANC.

We also found evidence of effect modification on the additive and multiplicative scales by partners’ education on the relationship between planned pregnancy and early ANC. This finding suggests that there were strong indications that the estimated joint effect of planned pregnancy on the additive or ratio scale with mothers whose partners had secondary or higher education was larger than the estimated effect of planned pregnancy with mothers whose partners had less than a secondary education. Although previous studies have shown that husband’s education was associated with early ANC [17, 36], our study represents an important contribution by further demonstrating that mothers whose partners had secondary or higher education were not only associated with early ANC, but that coverage of early ANC was further increased if such mothers had their pregnancies planned.

Our analysis also found that among mothers whose partners had a secondary or higher education, early ANC was more prevalent among employed mothers compared to unemployed mothers. A plausible explanation may be that employment does not only provide economic empowerment and autonomy to make sexual and reproductive health decisions [37, 38], but also enable women implement these decisions, which include the utilization of ANC services [39]. Our finding is consistent with previous studies in Ghana, Nigeria, Malawi, and Papua New Guinea, which found that working women were more likely to initiate early ANC compared to non-working women [13, 15, 40]. However, these findings were irrespective of whether the women had partners who had a secondary/higher education or not. The implication of our finding is that socio-economic inequalities and providing employment opportunities for women remains an important priority area to improve uptake of early ANC.

In this study, we also observed that among mothers whose partners had less than a secondary education, mothers who had teenage pregnancy were less likely to have early ANC compared to mothers who were 20 years or older. This finding may be due to the repercussions associated with teenage pregnancy. Women with teenage pregnancy are less likely to seek ANC due to the fear of social stigma [41]. Teenage pregnancy is also associated with late disclosure of pregnancy and unfriendly ANC services among some healthcare providers [42, 43], which can delay initiation of early ANC.

Without regard to the educational status of their partners, we found that early ANC was less prevalent among mothers who had teenage pregnancy compared to older mothers. This finding was in conformity with other studies [18, 44]. Our finding on higher prevalence of early ANC among mothers whose pregnancies were planned compared to those whose pregnancies were unplanned also aligned with previous studies [45–47].

This study had several strengths and limitations that should be acknowledged. Our study population was a convenient sample and therefore our findings may not be generalizable to “other” populations. Nonetheless, to the best of our knowledge this is the first study to show evidence of effect modification by partners’ education on the relationship between planned pregnancy and early ANC on both the additive and multiplicative scales. We also demonstrate how the relationship between employment status and early ANC, and between teenage pregnancy and early ANC, varied by partners’ level of education. Most of the variables in our study were also self-reported, but we expect recall bias to be similar among mothers who had early ANC and mothers who did not. In addition, early ANC was objectively verifiable using maternal and child health record book, so we expect that our outcome variable is less likely to be subject to recall bias. Another limitation is that our findings do not infer causality as this study was cross-sectional. There is also the potential for residual confounding as we adjusted for a limited number of confounding variables.

## Conclusion

This study found that mothers whose partners had secondary or higher education were associated with early ANC, and the relationship between mothers whose pregnancies were planned, and early ANC were modified by partners’ education on both the additive and multiplicative scales. In relation to women in Northern Ghana, we recommend that early ANC might be improved if programmes and policies focus on modifiable factors identified in our study.

## Data Availability

The dataset for this study is available from the corresponding author on reasonable request.

## Abbreviations

ANC: Antenatal care
aPR: Adjusted prevalence ratio
CIs: Confidence intervals
COVID-19: coronavirus disease 2019
FANC: Focused antenatal care
GEE: Generalized estimating equation
RERI: Relative excess risk due to interaction
STROBE: Strengthening the reporting of observational studies in epidemiology
WHO: World Health Organization
